# Antioxidant activity of tuberosin isolated from *Pueraria tuberose *Linn

**DOI:** 10.1186/1476-9255-7-47

**Published:** 2010-09-14

**Authors:** Nidhi Pandey, Yamini B Tripathi

**Affiliations:** 1 Department of Medicinal Chemistry, Institute of Medical Science, Banaras Hindu University, Varanasi-221005, India

## Abstract

Antioxidant activity of *Pueraria tuberose *DC, (PT) *Leguminosae (Fabaceae) *has already been reported by us and here an active compound has been isolated and its action on expression of iNOS protein has been explored by using LPS induced changes in attached rat peritoneal macrophage cell culture. The pure compound was isolated by column chromatography and its structure was characterized by spectral studies, which was identified as tuberosin (5 hydroxy 3,4,7,3',4' pentamethoxy flavone). Its antioxidant capacity was determined and compared with alcoholic extract as EC_50 _value for scavenging potential towards pre-generated monocation ABTS* radical, superoxide radicals, hydroxyl radicals, metal chelation property and on lipid peroxidation. Further, rat peritoneal macrophages were isolated, cultured and the attached macrophages were exposed to lipopolysaccharide (LPS) with different concentrations of tuberosin (pretreatment for 30 min). After 17 h the released NO content, in culture supernatant, was indirectly estimated as accumulated nitrite by Griess reagent. To understand the mechanism of action, the extent of expression of inducible nitric oxide synthase genes, the iNOS protein was assessed in macrophage lysate by using its antibody on western blot analysis. Tuberosin significantly scavenged all the species of FRs, described above and it also inhibited the LPS induced release of NO and amount of iNOS protein in macrophages. All the changes were significant and concentration dependent. Thus it could be suggested that tuberosin, is one of the active principles of *Pueraria tuberose*, which directly scavenges various species of Free radicals (FRs) and also inhibits LPS induced inflammatory changes in macrophages.

## Background

In recent years, phyto-medicine is in great demand as food supplement for age related chronic diseases, because of their multi-targeted action and lesser side effects [1] In fact, these diseases are associated with generation of excessive free radical (FR) [2] and associated inflammation [3] and these herbal products are rich in polyphenols, specially flavones and tannins. Therefore, search for potent antioxidants with anti-inflammatory potential has always been in demand. In various countries, these herbs are used as a component of their alternative system of medicine [4] and in Ayurveda, an Indian system of medicine, medicinal plants are well documented for their therapeutic claims, with records of long clinical use, for prevention and management of several metabolic disorders [5].

*Pueraria tuberosa *Linn (PT), *Leguminosae (Fabaceae)*, known as Bidaarikand [6] is an extensive perennial climber, with palmately arranged leaves, blue colored flowers and half inches thick bark [7], growing throughout tropical parts of India, mostly in moist regions, monsoon forests and coastal tracts. Its tuberous root, which is brown in color and slightly curved, is in clinical use for rejuvenation therapy. Its microscopic picture reveals the presence of prismatic calcium crystals and tanniniferous cells. It's major chemical constituents include flavones [C-glycoside (5,7,3',5'-tetrahydroxy-4'-methoxyflavone-3'-O-α-Lrhamnopyranosyl1→3-O-β-D-galactopyranoside)], Isoflavones (Puerarone), Coumstan (Tuberostan, Puerarostan) [8], Epoxychalcanol [Puetuberosanol], (3'-hydroxy-4'-phenoxy-α,β-epoxychalcan-α'ol)] [9], Pterocarpanoids [Hydroxytuberosin, Anhydroxytuberosin (3-O-methylanhydrotuberosin)] [10], and Tuberosin [11]. The powder of PT root-tubers are in clinical use as anti-aging and also as tonic, aphrodisiac, demulcent, lactagogue, purgative, cholagogue and also in scorpion sting. Besides, it is also useful in emaciation of children, debility and poor digestion [6,7]. Other investigators have reported it for skin care, as anti-fertility [12]. One of its phytochemical, purerin, has been associated with anti-diabetic property [13].

The presence of free transition metals in the biological system leads to excessive generation of free radicals [14]. However when the natural antioxidant enzymes are not sufficient to scavenge these active FRs, then their unusual longer persistence in the cell, causes peroxidation of cellular lipids and proteins, which results to damage of cell-organelles. Further these oxidized macromolecules behave as foreign proteins and affect the immune system. They may activate the inflammatory cascade, resulting in initiation of various degenerative diseases and autoimmune disorders [15]. Therefore, these antioxidants have variety of other biological responses, because of their indirect influence on inflammatory and immunity pathway. To name a few, these includes eugenol, gallic acid and quercetin [16-19].

As we have already reported the antioxidant property of PT tuber extract [20], so here its active principle has been isolated and the role of inflammation has been explored. Since alcoholic fraction of PT tuber had shown most potent FR scavenging potential, therefore it was subjected to column chromatography and the isolated compounds were tested for their antioxidant potential and one of its most active compounds was characterized by spectral analysis. Its property was compared with its mother extract in terms of their EC_50_. Further, its anti-inflammatory property was explored by monitoring its inhibitory effect on LPS (Lipopolysaccharide) induced expression of inducible nitric oxide synthase (iNOS) and release of nitric oxide (NO) in the culture supernatant, by attached rat peritoneal macrophages culture.

## Methods

### Material

2,2'-azinobis-3-ethyl benzothiazoline-6-sulfonic acid (ABTS*), Deoxyribose, were purchased from Sigma Aldrich Co. USA. Nitrobluetetrazolium (NBT), Riboflavin, L-methionine, thiobarbituric acid, Ethylenediamine tetra acetic acid (EDTA) were purchased from Hi-Media Ltd, ferric chloride anhydrous (FeCl_3_) ascorbic acid, trichloro acetic acid, potassium persulfate Vitamin C were purchased from Merck Ltd. All the other reagents were of analytical grade.

### Isolation and characterization of Tuberosin

The root-tubers of *Pueraria tuberose *were purchased from local market and its authenticity was rechecked on pharmacognostical parameters. Its voucher specimen was persevered in the dept. (No. YBT/MC/12/1-2007). The dried root-tuber -powder was successively extracted with hexane and then with ethanol in a soxhlet extractor. The solvent free alcoholic extract (yield-12-18% w/w) was saved for column chromatography. 8 g of this extract was separated over silica gel column (80 × 4 cm) and eluted with organic solvent with increasing polarity. The ethyl acetate fraction was subjected to re-chromatography on a smaller silica gel column (30 × 1.5 cm) by using benzene:ethyl acetate (7:3) as elution solvent. The isolated compound was re-crystallized from benzene, which furnished white crystals, m.p. 271-272°C. Its purity was confirmed by thin layer chromatography on silica gel G plate, where it showed single spot of Rf value 0.45 with solvent, Benzene: Chloroform (6:4). The spectral data of the isolated compound (UV, IR and NMR) were compared with the data of other compounds, isolated from PT extract and reported in the literature [11]. Based on similarity, this biologically active isolated compound was identified as 5 hydroxy 3,4,7,3',4' pentamethoxy flavone (Tuberosin).

### 2. Assay of antioxidant property

#### a. ABTS* radical scavenging activity

ABTS* radical scavenging activity of tuberosin was determined according to Re et al. [21], where ABTS* radicals were pre-generated by mixing solutions of ABTS* (14 mM) and potassium persulphate (4.9 mM). After mixing different concentrations of the test compound with the ABTS* solution, the reduction in degree of absorbance was recorded at 734 nm.

#### b. Lipid peroxidation assay

Lipid Peroxidation assay was carried out by modified method to measure thiobarbituric acid-reactive substances (TBARS) [22], where FeSO_4 _was used to induce lipid peroxidation in egg yolk homogenates [23]. The pink colour, developed after heating the reaction mixture in water bath for 1 h, was read at 532 nm.

#### c. Superoxide radical scavenging property

Superoxide radical scavenging property was assessed by monitoring the capacity of test compounds to scavenge instantly generated superoxides, through riboflavin mediated photosensitive reaction. The added NBT solution reacted with superoxide radicals and rate of formation of its coloured product was monitored at 560 nm [24].

#### d. Hydroxyl radical scavenging property

Similarly, hydroxyl radical scavenging potential was measured by Non Site-specific hydroxyl radical-mediated 2-deoxy-D-ribose degradation. Here, the reaction was carried out in presence of FeCl_3 _and EDTA. Here, its complex reacted with H_2_O_2 _in presence of ascorbic acid to produce OH radicals, which degraded the deoxyribose to a coloured end product, which was monitored at 532 nm. Finally to assess the metal chelating property of the test material, the Site-specific hydroxyl radical-mediated 2-deoxy-D-ribose degradation was monitored, where the above reaction was carried out in absence of EDTA. The difference in the readings of the above 2 reactions were considered as degree of metal chelation [25].

### 3. Effect on NO production

Inbred male rats of Charls foster (CF) strain of matched age and weight were purchased from the central animal house of Institute of Medical Sciences and acclimatized in our laboratory conditions for 7 days. On the experimental day, the rats were anaesthetized by injecting ketamine and 10 ml of sterile ice-cold phosphate buffer saline, devoid of calcium and magnesium ions was injected in to the peritoneal cavity to each rat, through a syringe [26]. The abdomen was squeezed for 5 min, and then the peritoneal fluid was aspirated out. It was centrifuged and the cell pellet was washed 2 times with serum free RPMI-1640 media to harvest the macrophages. This cell preparation was finally suspended in a known volume of complete RPMI-1640 media supplemented with 5% fetal calf serum (FCS). The isolated macrophages were counted by trypan blue exclusion method in haemocytometer and appropriately diluted to have 1 × 10^4 ^cells in 200 μl, which was taken in each cavity of 96 well culture plate. The plate was incubated for 2 hr at 37°C in 5% CO_2 _atmosphere to attach the living macrophages [27,28] and then culture supernatant was replaced with fresh complete media. The attached macrophages were used for various experiments as described in respective tables. All tests were carried out in triplicate. In one set only drug vehicle (0.1% DMSO) was added, in another set, quercetin was added as positive control and in test wells, different concentrations of tuberosin were added. After pre-incubation for 30 min, LPS (20 ng/ml) was added to each well, mixed and incubated overnight for 17 hours to induce nitric oxide (NO) production. Next day, accumulated nitrite in the culture supernatant was monitored by using Griess reagent [29] (1% sulfanilamide/0.1% naphthalene diamine dihydrochloride 2.5% H_3_PO_4_). Absorbance was read at 550 nm in an ELISA plate reader (Multiscan). It is an indirect method to measure the accumulated nitrite in the culture supernatant, which reflects the concentration of released nitric oxide. The EC_50 _value of isolated compound (concentration of sample required to inhibit 50% response of LPS for NO production) for each parameter were determined by statistical formula, given below in the method section.

### 4. Effect on iNOS expression by Western blot Analysis

After removing the culture supernatant for nitrite estimation, the attached macrophages were washed with PBS and then lysed by adding 200 μl lysis buffer (20 mM Tris-Buffer (pH = 7.4), containing 0.25 sucrose, EDTA (1 mM), PMSF (100 *μ*g ml^−1^), aprotinin (10 *μ*g ml^−1^), leupeptin (10 *μ*g ml^−1^). The protein of this cell lysate was estimated by Bradford method [30] and its 20 *μ*g protein was run in each lane on 8% sodium dodecyl sulphate-polyacrylamide gel electrophoresis (SDS-PAGE) [31]. The separated protein bands were transferred to nitrocellulose membrane by electro-blotting, washed with TBS (Tris-buffered saline) containing 0.05% (v/v) Tween 20 and blocked with 5% (wt/vol) dried non-fat milk in TBS for 2 hrs. Finally, the blot was incubated with rabbit polyclonal anti-iNOS antibody (SC650, Santa Cruz Biotechnology, 1/1000 in TBS-Tween-20 buffer) at 4°C overnight and visualized by alkaline phosphatase-conjugated anti-rabbit IgG as the secondary antibody. DAB (diamminobenzidine) was used as substrate [32]. The intensity of bands was analyzed by image analyzer-2254. The equal loading of sample in each lane was confirmed by monitoring the expression of ß-actin.

### 5. Statistics

All data were expressed as means ± SD. Pearson's correlation analysis (SPSS 7.5 for Windows, SPSS Inc.) was used to test for the significance of relationship between the concentration and percentage inhibition at a *p *< 0.05 significance level. The EC_50 _of for different parameters were calculated by using the following formula

$${\text{Y}}_{50}=\text{A}+\text{BX}$$

***Where*, **A = Mean of × - B (predicted Y value=, 50%)

$$B=\frac{\sum \text{X}.\text{Y}-(\sum \text{X})(\sum \text{Y})/\text{N}}{\sum {\text{Y}}^{2}-{\left(\sum \text{Y}/\text{N}\right)}^{2}}$$

X = independent variable (Concentration of Drug)

Y = dependent variable (% inhibition)

## Results

### (1) Characterization of Tuberosin

**Figure 1:**
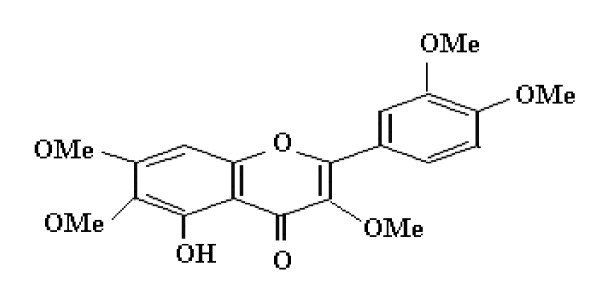
**Structure of tuberosin**.

The spectral data of the isolated compound for UV, IR, ^1^H-NMR, and ^13^C NMR (Table 1) were compared with the data available in the literature and based on the similarity, the isolated compound was identified as was tuberosin (figure 1).

**Table 1:** Analytical data of isolated compound (Tuberosin; 5hydroxy 3,6,7,3'4' pentamethoxy flavone)

Melting point	271-72°C
TLC pattern	Solvent system: benzene:ethyl acetate (7:3) RF value: 0.45

UV(MeOH)	(log ε): 255(4.26), 274 (4.18) and 346 nm (4.21)

IR (KBr) cm^-1^	3480, 1664 and 1559

^1^H NMR (CDCl_3_)	δ 12.62 (1 H, s, O - H), 7.75 (2 H, m, 2' - H and 6' - H), 7.01 (1 H, d, J = 9.0 Hz, 5' - H), 6.51 (1 H, s, 8 - H), 3.98 (9 H, s, 3 × OCH_3_), 3.93 (3 H, s, OCH_3_) and 3.87 (3 H, s, OCH_3_).

^13^C NMR	δ 158.7 (C - 2), 132.4 (C - 3), 178.8 (C - 4), 155.7 (C-5), 138.8 (C-6), 152.7 (C-7), 90.3 (C- 8), 151.5 (C-9), 106.6 (C-10), 122.9 (C-1'), 111.7 (C-2'), 148.9 (C-3'), 152.2 (C-4'), 111.6 (C-5'), 122.1 (C-6'), 60.7 (6-OCH_3_), 56.1 (7-OCH_3_), 60.1 (3-OCH_3_), 56.2 (3' - OCH_3_) and 55.9 (4' - OCH_3_).

### (2). ABTS* assay

Tuberosin scavenged the pre-generated ABTS* radicals in concentration-dependent manner, with EC_50 _values as 70 ng/ml, which was lower as compared to its mother extract (alcoholic fraction of PT- 320 μmug/ml). The difference was in the range of 44.71 fold (Table 2).

**Table 2:** Effect of tuberosin on pre-generated ABTS* radical and superoxide radical scavenging property

Concentration of tuberosin (μmuM)	% decrease in absorbance at 734 nm (mean ± S.D.) for ABTS* radical scavenging	% decrease in absorbance at 560 nm (mean ± S.D.) for SO radical scavenging
50	8.42 ± 0.99*	5.78 ± 0.46**

125	24.09 ± 0.33*	18.98 ± 0.76**

200	45.16 ± 0.89*	27.89 ± 0.55**

250	60.00 ± 1.05*	54.78 ± 0.87**

375	77.25 ± 1.06*	67.11 ± 0.77**

500	93.08 ± 0.63 *	83.44 ± 0.63**

775	97.24 ± 0.89*	97.24 ± 1.22**

EC_50_	198.67 μmuM	205.11 μmuM

	EC_50 _of Vit C- 220 μmuM,	EC_50 _for quercetin- 0.60 μmuM

n = 3, Level of significance: p* < 0.1 and p** < 0.001

### (3). Superoxide scavenging assay

Tuberosin also scavenged the instantly generated superoxide radicals in a concentration-dependent manner with EC_50 _value at 156 μmug/ml (Table 2), which was 1.5 times lower than it's alcoholic mother extract (240 μmug/ml).

### (4). Lipid Peroxidation Assay

There was significant and concentration-dependent inhibition by tuberosin on FeSO_4 _induced lipid peroxidation (Table 3). Tuberosin had 7.95 fold lower EC_50 _value (98 μmug/ml) as compared to the alcoholic extract of PT (780 μmug/ml).

**Table 3:** Inhibition of lipid peroxidation induced by FeSO_4 _using egg yolk homogenates

Concentration of tuberosin (mM)	Absorbance at 532 nm (mean ± SD)	% decrease in absorbance (mean ± SD)
Blank	0.380	--

control	0.355	--

12	0.320 ± .021*	7.08 ± 1.30

18	0.283 ± .020*	17.89 ± 1.00

25	0.248 ± .018**	27.98 ± 1.42

30	0.198 ± .015**	42.74 ± 0.96

40	0.131 ± .010**	62.05 ± 1.50

45	0.073 ± .002**	78.76 ± 1.16

50	0.049 ± .001**	85.83 ± 1.30

n = 3 EC_50 _of tuberosin- 49.22 mM, EC_50 _for quercetin- 0.60 μmuM^@^

Level of significance: p* < 0.1 and p** < 0.001. ^@^Reference 18

### (5). Non-site specific Hydroxyl radical scavenging assay (With EDTA)

Tuberosin was found to be the more potent hydroxyl radical scavenger with EC_50 _values of (32 μmug/ml), which was 9.6 time lower than it's alcoholic fraction (EC_50 _310 μmug/ml) (Table 4).

**Table 4:** Effect of tuberosin in the deoxyribose assay in the presence of EDTA (non-site specific) to assess the Hydroxyl radical scavenging activity and absence of EDTA (site specific) to assess metal chelation property

Concentration of tuberosin (mM)	Absorbance at 532 nm (mean ± S.D)		% decrease in absorbance (mean ± SD)	
	
	(Non site specific)	(Site specific)	(Non site specific)	(Site specific)
Normal	0.310 ± .018	0.515 ± .028	--	--

Blank	0.288 ± .017	0.485 ± .022	--	--

0.25	0.280 ± 0.014*	0.400 ± .024*	2.60 ± 0.94	17.54 ± 1.25

0.50	0.256 ± .015*	0.356 ± .021**	11.22 ± 0.97	26.54 ± 1.15

0.75	0.235 ± .013**	0.311 ±.020**	18.35 ± 0.88	35.82 ± 0.91

1.00	0.199 ± .012**	0.271 ± .018**	30.73 ± 1.08	44.08 ± 0.96

1.25	0.156 ± .011**	0.190 ± .011**	45.94 ± 0.94	60.80 ± 0.94

1.80	0.125 ± .010**	0.119 ± .008**	56.72 ± 1.14	75.51 ± 0.99

2.50	0.092 ± .007**	0.01 ± .001**	67.94 ± 0.67	98.04 ± 0.62

n = 3; EC_50 _of tuberosin: Non site specific assay = 1.14 mM and site specific assay = 0.918 mM; EC_50 _(μmuM) for quercetin - Non site specific assay 0.80 and site specific assay- 0.50; Level of significance: p* < 0.1 and p** < 0.001.

### (6) Site specific Hydroxyl radical scavenging assay (Without EDTA)

Further in the case of *Site specific Hydroxyl radical scavenging assay *(without EDTA), EC_50 _values of tuberosin was at 28 μmug/ml, which was lower than the value obtained in case of non site specific reaction (described above), suggesting its additional role as metal chelation (Table 4).

### (7) Effect of tuberosin on LPS induced NO production and iNOS-protein expression in macrophages

**Figure 2:**
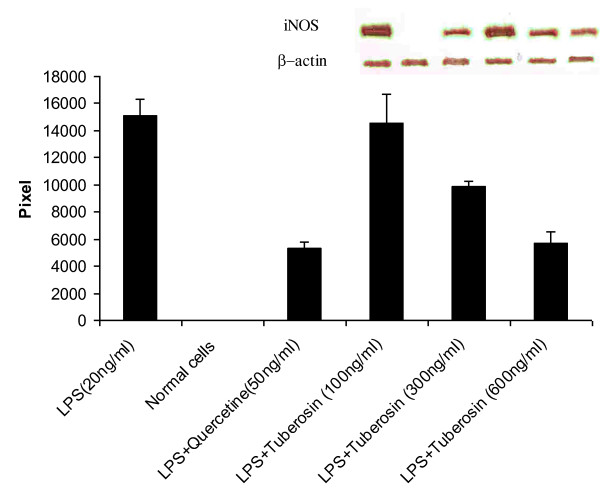
**Effect of different concentrations of Tuberosin on LPS induced iNOS expression in attached rat peritoneal macrophages**. The macrophages were pretreated with quercetin and tuberosin as given below for 30 minutes and then LPS was added (20 ng/ml) and incubated for 17 hrs. The normal cells were exposed to 0.1% DMSO without any LPS. Lane-1: LPS(20 ng/ml),Lane-2: Normal cells. Lane3:LPS+Quercetine(50 ng/ml), Lane4:LPS+Tuberosine(100 ng/ml), Lane-5: LPS+Tuberosine(300 ng/ml), Lane-6: LPS+Tuberosine(600 ng/ml). The bars depict densitometric analysis of western blot (given in the inset). This picture represents one out of total three experiments carried out separately.

Tuberosin significantly inhibited LPS induced release of nitric oxide (NO) by macrophages in concentration-dependent manner (Table 5). It also inhibited the accumulation of iNOS proteins in the attached macrophages (Figure 2).

**Table 5:** Effect of tuberosin on LPS induced NO production and iNOS expression by attached rat peritoneal macrophages.

Concentration of tuberosin (ng/ml)	NO production (μg/10^4^cells)	Pixel value of iNOS bands in western blot
Normal Cells	10.11 ± 1.043	-

Only LPS (20 ng/ml)	39.89 ± 1.983	16023

LPS(20 ng/ml)+Tuberosin(ng/ml)		-

100	38.09 ± 1.933	15878

200	36.09 ± 1.862**	-

300	27.02 ± 1.698**	10678

400	21.16 ± 1.829*	-

500	13.04 ± 1.904*	-

600	10.09 ± 1.898*	5082

LPS + Quercetin(50 ng/ml)	9.98 ± 1.041	4223

EC_50 _of tuberosin	399.68 ng/ml	-

EC_50 _of quercetin	190 ng/ml	-

Values were significant (p* < 0.1, p** < 0.001) when compared with experimental control.

## Discussion

Various pure isolated phytochemicals or plant extracts having natural cocktail of various poly-phenolics, have shown antioxidant and anti-inflammatory property [33,34]. They are also in use for the management of age related chronic diseases such as diabetic complications [35], atherosclerosis [36] and inflammation [37], as food supplement or as add-on therapy with conventional medicine.

The powder of PT root-tubers are already in clinical use by Ayurvedic physicians of Indian system of medicine [6], but neither its mechanism of action nor the active principle for its antioxidant and anti-inflammatory property has been explored so far. Interestingly, our data has helped in characterizing the isolated compound as tuberosin, which has already been reported [11], but no biological activity related to LPS induced changes, has been available in the literature.

Tuberosin has exhibited direct FR trapping capacity in a chemical reaction system, however, variability in its potency towards various free radical species, could be because of the difference in the electron potential of these free radical species [38]. Further, the Fe induced lipid peroxidation in presence of ascorbic acid, is an example non-enzymatic process (Fe^++^/ascorbic acid), therefore, the anti-lipid-peroxidative property of tuberosin, described above, indicates its total antioxidant capacity. As it has also shown metal chelation property along with direct FR trapping property, therefore the net response of inhibition towards lipid peroxidation could be a combined effect of these 2 responses.

Tuberosine has shown lower EC_50 _value on all tested parameters than its mother alcoholic extract, which suggests its higher potency, and therefore it could be considered as its active principle. However, it has been found to be significantly less potent than quercetin, which could be because of structural difference in these two compounds. It has been documented earlier that number and position of hydroxyl groups in the flavones ring, regulates its antioxidant potential and the presence of 3-OH makes the compound more potent than that of 5-OH group [39]. From the structural comparison of these 2 compounds, it is clear that tuberosine has 5-OH group, where as quercetin has 3-OH group. Thus, the higher potency of quercetin over tuberosin could be explained.

Measurement of inhibitory property of a test compound against LPS induced NO release is one of the standard models to explore anti-inflammatory potential of any test drug. LPS is known to induce iNOS through activation of NF-kB and this process involves free radicals (FR) in its early steps, just after interacting with its Toll-like receptor (TLR) [40,41]. Therefore, free radical scavengers have been reported earlier to inhibit this process and our data has also shown concentration-dependent inhibition of LPS induced NO release. This trapping capacity of tuberosin, for variety of free radical species and also for metal chelation property has been found in our *in vitro *testing on a chemical test model. Thus, it could be suggested that tuberosin might be acting on the initial steps of the signaling cascade of LPS induced NO production, but it is still not clear, whether it is directly inhibiting the activity of iNOS enzyme or it is suppressing the synthesis of this enzyme.

To target this question, we explored the effect of tuberosin on iNOS protein in macrophages, when exposed to LPS. Interestingly, our data show that tuberosin significantly inhibited the iNOS protein in western blot analysis. The results suggested that tuberosin is inhibiting the expression of iNOS genes, as amount of iNOS proteins was significantly lower in tuberosin pre-treated cells in concentration dependent manner.

## Conclusion

From the above experimental results, it could be suggested that tuberosin is one of the active principles of *Pueraria tuberose *for its claimed antioxidant property. The tuberosine has direct scavenging potential for variety of free radicals with preference to ABTS* radicals followed by hydroxyl radicals and then superoxide radicals. It has additional metal chelation property. Tuberosin has potential to inhibit LPS induced NO production in concentration-dependent manner, which is due to inhibition in the expression of iNOS proteins.

## Competing interests

The authors declare that they have no competing interests.

## Authors' contributions

NP carried out the experimental works. YBT conceived of the study, and participated in its design, discussion of results, over all coordination and wrote the manuscript. All authors read and approved the final manuscript.
